# How do clinical researchers’ and patients’ preferences influence study hypotheses and reported outcome results for clinical randomised controlled trials? A critical appraisal

**DOI:** 10.1186/1745-6215-16-S1-P34

**Published:** 2015-05-29

**Authors:** Paola Rosati, Roberto D’Amico, Gabriella Ricciotti, Giuseppina Testa, Rita Inglese, Ferruccio Giustini, Ersilia Fiscarelli, Marco Zazza, Cecilia Carlino, Valerio Balassone, Roberto Fiorito, Franz Porzsolt

**Affiliations:** 1 G.A.L.I.L.E.O. Gruppo Apprezzamento Letteratura e Implementazione Livelli di Evidenza in Ospedale, Bambino Gesù Children’s Hospital IRCCS, Rome, 00165, Italy; 2 Unit of Clinical Epidemiology, Bambino Gesù Children’s Hospital IRCCS, Rome, 00165, Italy; 3 Italian Cochrane Center, Department of Diagnostic, Clinical and Public Health Medicine, University of Modena and Reggio Emilia, Modena, 41124, Italy; 4 Faculty of Medicine and Surgery, La Sapienza University, Rome, 00185, Italy; 5 General Surgery and Transplantation Department, University Tor Vergata, Rome, 00133, Italy; 6 Health Care Research, General and Visceral Surgery, University Hospital Ulm, Germany President, Institute of Clinical Economics (ICE) e.V., Ulm,.89070, Germany

## Background

Studies designed to promote unbiased research increasingly show that human preferences exert a major influence on randomised controlled trials (RCTs) [1-3]. More information is needed on how preferences influence clinical trial design and conduction [4-6]. To fill the information gap between what researchers seek and report and what patients want [7,8], in this study we investigated how researchers’ and patients’ preferences influence study hypotheses and outcome results of published clinical RCTs. Because conventional critical appraisal seemed inappropriate for addressing our research question, in this pilot study we developed a novel assessment method and applied it in an RCT sample.

## Methods

We collected 20 unselected and consecutive RCTs published in a high impact paediatric journal from July to November 2013. Two experienced reviewers identified the following five domains and a grading method to score discrepancy between what author’s state in clinical trial registries (CTRs) and report in published RCTs: reported funding (1 point), study hypotheses, information on patients enrolled and study conduction (3 points); primary and secondary outcomes, early study completion, and upgrading or downgrading outcome results (5 points). Higher scores implied marked discrepancy. Two reviewers then independently applied the method on the RCT sample by mapping and coding information for the domains identified and reported discrepancies by comparing CTRs and RCTs (Table S1, Additional file 1).

## Results

Of the 20 RCTs collected and CTRs compared, 14 studies had high total preference discrepancy scores (7 scored 10-12, and 7 scored 16 or more) and 4 had discrepancy in declaring funding. In 12 studies researchers completed the study early and in 8 studies they downgraded or upgraded outcomes. Only 5 CTRs were updated but they neglected to include published RCT results. Only in 5 CTRs, dataset supervisors indicated the RCT URL. None of the 20 RCTs allowed us to assess patients’ preferences (no information reported for non-response and refusal). No difference was found in discrepancy scores among the five CTR databases.

## Conclusions

The high discrepancy scores obtained by comparing what researchers stated in CTRs and published in RCTs suggest possible misconduct. Patients’ preferences during RCT enrolment and conduction remain undetectable owing to the lack of targeted protocols to elicit this issue. These results, if confirmed in further studies, should prompt international regulation developers [2] to encourage researchers to explore patients’ preferences as a strategy to enhance informed decision-making and to improve reporting in RCTs and CTRs.

## Supplementary Material

Additional file 1 Click here for file

## References

[B1] King M Nazareth I Lampe F Bower P Chandler M Morou M Impact of participant and physician intervention preferences on randomized trials: a systematic review JAMA 2005 293 1089 99 10.1001/jama.293.9.1089 15741531

[B2] WHO Statement on public disclosure of clinical trial results Call for public consultation 2014 http://www.who.int/ictrp/results/en/

[B3] Walker E Nowacki AS Understanding equivalence and noninferiority testing J Gen Intern Med 2011 26 192 196 10.1007/s11606-010-1513-8 20857339 PMC3019319

[B4] Stengel D Sehouli J Porzsolt F Porzsolt F, Kaplan RM Are the results of randomized trials influenced by preference effects? Part I. Findings from a systematic review Optimizing Health-Improving the Value of Healthcare Delivery 2006 New York: Springer 265 291

[B5] Porzsolt F Stengel D Porzsolt F, Kaplan RM Are the results of randomized trials influenced by preference effects? Part II. Why current studies often fail to answer this question Optimizing Health- Improving the Value of Healthcare Delivery 2006 New York: Springer 292 297

[B6] Ioannidis JPA Greenland S Hlatky MA Khoury MJ Macleod MR Moher D Increasing value and reducing waste in research design, conduct and analysis Lancet 2014 383 166 75 10.1016/S0140-6736(13)62227-8 24411645 PMC4697939

[B7] Wade J Donovan JL Lane JA Neal DE Hamdy FC It’s not just what you say, it’s also how you say it: opening the ‘black box’ of informed consent appointments in randomised controlled trials Soc Sci Med 2009 68 2018 2028 10.1016/j.socscimed.2009.02.023 19364625

[B8] Mills N Donovan JL Wade J Hamdy FC Neal DE Lane JA Exploring treatment preferences facilitated recruitment to randomized controlled trials J Clin Epidemiol 2011 64 1127 1136 10.1016/j.jclinepi.2010.12.017 21477994 PMC3167372

