# Supplementary material for: How do clinical researchers’ and patients’ preferences influence study hypotheses and reported outcome results for clinical randomised controlled trials? A critical appraisal
Source: Trials. 2015 May 29;16(Suppl 1):P34. doi: 10.1186/1745-6215-16-S1-P34 (PMC4460712; doi:10.1186/1745-6215-16-S1-P34)
Supplement: Additional file 1 [file 1745-6215-16-S1-P34-S1.pdf]

Table 1. Reviewers' estimated discrepancy score between issues reported by authors in clinical trials registries and what they report in published randomised controlled trials

National Registries and number of trial registration	First author and reference in RCTs published in *Pediatrics*	Total preference discrepancy score High 10-20 Medium 5-9 Low <5
International Standard Randomised Controlled Trial Number Register, BioMed Central, UK ISRCTN 31707342 www.controlled-trials.com	McCarthy LK et al. 132, 1, e135-e141 July 2013	0 Low
US National Controlled Trials NCT 01822626 https://clinicaltrials.gov	Davoli A.M. et al. 132, 5, e1236-e1245 Oct 2013	2 Low
International Standard Randomised Controlled Trial Number Register, BioMed Central, UK ISRCTN 59061709 www.controlled-trials.com	McCarthy LK et al. 132, 2, e389-e395 Aug 2013	2 Low
Australian New Zealand Clinical Trial Registry ACTRN 12608000056392 https://www.anzctr.org.au	Daniels LA et al. 132, 1, e109- e118 July 2013	3 Low
Netherlands Trial Registry NTR1613 www.trialregister.nl	Van der Veek et al. 132, 5, e1163-e1172 Nov 2013	3 Low
US National Controlled Trials NCT 00409448 https://clinicaltrials.gov	Kurowski et al. 132, 1, e158-e166 Jul 2013	6 Medium
US National Controlled Trials NCT00548379 https://clinicaltrials.gov	Aluisio A.R. et al. 132,4, e832-e840 Oct 2013	10 High
International Standard Randomised Controlled Trial Number Register, BioMed Central, UK ISRCTN 72635512 www.controlled-trials.com	Field 132, 5, e1247-e1256 Nov 2013	12 High
US National Controlled Trials NCT 01307293 https://clinicaltrials.gov	Shaw RJ et al. 132, 4, e886-e894 Oct 2013	12 High
International Standard Randomised Controlled Trial Number Register, BioMed Central, UK ISRCTN 03981121 www.controlled-trials.com	Wake M et al. 132, 4, e895-e904 Oct 2013	12 High
Clinical Trials Registry India CTRI/2010/091/001417	Malik A et al. 132, 1, e46-e52 July	12 High

www.ctri.nic.in	2013	
US National Controlled Trials<br>NCT 01351064<br>https://clinicaltrials.gov	Carroll A.E. 132, 3,<br>e623-e 629 Sept<br>2013	12 High
Netherlands Trial Registry<br>NTR 2061 www.trialregister.nl<br>and<br>Australian New Zealand Clinical Trial Registry<br>ACTRN 12610000230055<br>https://www.anzctr.org.au	Kamlin COF et al.<br>132, 2, e381-e388<br>Aug 2013	12 High
US National Controlled Trials<br>NCT 01403623<br>https://clinicaltrials.gov	Leadford AE et al.<br>132, 1, e128-e134<br>Jul 2103	16 High
US National Controlled Trials<br>NCT 01810978<br>https://clinicaltrials.gov	Dilli D. et al. 132, 4,<br>e932-e938 Oct 2013	17 High
US National Controlled Trials<br>NCT 00334737<br>https://clinicaltrials.gov/	Ohls RK et al. 132,<br>1, e119-e127 Jul<br>2013	17 High
US National Controlled Trials<br>NCT 01065272<br>https://clinicaltrials.gov	Alansari K. et al.<br>132,4, e810-e816<br>Sept 2013	17 High
Australian New Zealand Clinical Trial Registry<br>ACTRN 12612000976886<br>https://www.anzctr.org.au	McIntosh CG et al. 13<br>2, 326-331 Aug 2013	17 High
US National Controlled Trials<br>NCT 00551642<br>https://clinicaltrials.gov	Durrmeyer X et al.<br>132,3, e695-e703<br>Sept 2013	18 High
US National Controlled Trials<br>NCT 01604460<br>https://clinicaltrials.gov	Belsches et al 132,<br>3, e656-e661 Sept<br>2013	18 High
